# Supplementary material for: Prognostic significance of severe coronary microvascular dysfunction post-PCI in patients with STEMI: A systematic review and meta-analysis
Source: PLoS One. 2022 May 16;17(5):e0268330. doi: 10.1371/journal.pone.0268330 (PMC9109915; doi:10.1371/journal.pone.0268330)
Supplement: S1 File — (DOCX) [file pone.0268330.s002.docx]

**Supplementary material**

**S2- Exact search terms used for systematic reviewing in Medline, Pubmed and Google scholar.**

Key terms : (("myocardial"[All Fields] AND "infarction"[All Fields]) OR ("acute"[All Fields] AND "coronary"[All Fields] AND "syndrome"[All Fields]) OR ("percutaneous"[All Fields] AND "coronary"[All Fields] AND "intervention"[All Fields]) OR "angina"[All Fields] OR "revascularization"[All Fields] OR "angioplasty"[All Fields] OR ("coronary"[All Fields] AND "narrowings"[All Fields]) OR ("coronary"[All Fields] AND "stenoses"[All Fields]) OR ("coronary"[All Fields] AND "stenosis"[All Fields]) OR ("coronary"[All Fields] AND "artery"[All Fields] AND "disease"[All Fields])) AND (("coronary"[All Fields] AND "flow"[All Fields] AND "reserve"[All Fields]) OR ("coronary"[All Fields] AND "flow"[All Fields] AND "velocity"[All Fields]) OR ("microcirculatory"[All Fields] AND "resistance"[All Fields]) OR ("microvascular"[All Fields] AND "resistance"[All Fields]) OR ("coronary"[All Fields] AND "physiological"[All Fields] AND "indices"[All Fields]) OR ("reactivity"[All Fields] AND ("adenosine"[All Fields] OR "acetylcholine"[All Fields]))) AND ("prognosis"[All Fields] OR "prognostic"[All Fields] OR "event"[All Fields] OR "outcome"[All Fields] OR "death"[All Fields] OR "safety"[All Fields] OR "complications"[All Fields] OR "mortality"[All Fields] OR "endpoint"[All Fields] OR "endpoints"[All Fields] OR "adverse"[All Fields] OR "events"[All Fields] OR ("clinical"[All Fields] AND "significance"[All Fields])).
