# Supplementary figures and images for: Prognostic significance of severe coronary microvascular dysfunction post-PCI in patients with STEMI: A systematic review and meta-analysis
Source: PLoS One. 2022 May 16;17(5):e0268330. doi: 10.1371/journal.pone.0268330 (PMC9109915; doi:10.1371/journal.pone.0268330)

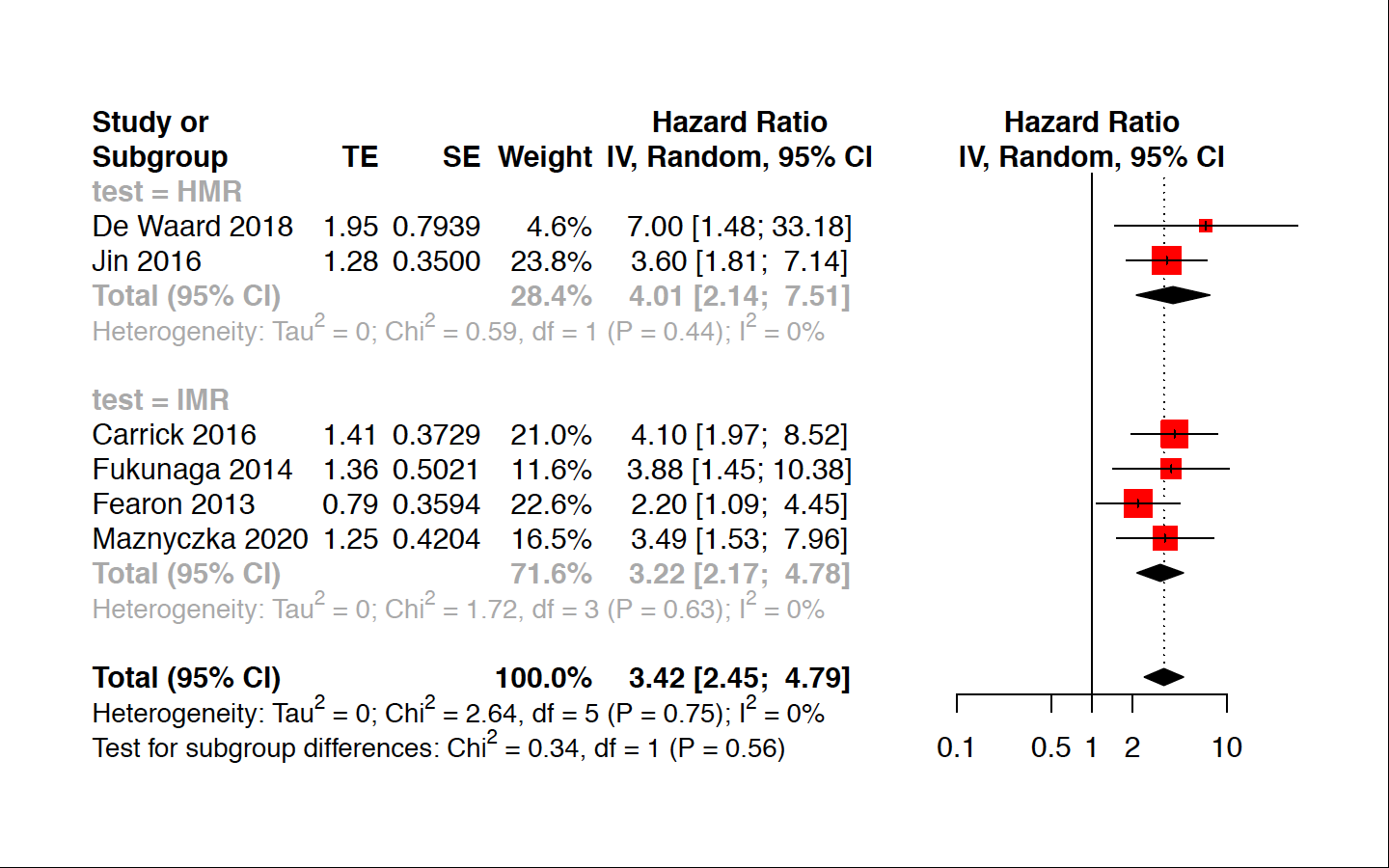

Supplement: S1 Fig — There was no difference between the two methods to assess CMV and the outcomes of interest (p = 0.56). (TIFF) [file pone.0268330.s003.tiff]
