# Supplementary material for: Prognostic significance of severe coronary microvascular dysfunction post-PCI in patients with STEMI: A systematic review and meta-analysis
Source: PLoS One. 2022 May 16;17(5):e0268330. doi: 10.1371/journal.pone.0268330 (PMC9109915; doi:10.1371/journal.pone.0268330)
Supplement: Attachment — Submitted filename: Answer metaanalysis 06 03 2022.docx [file pone.0268330.s004.docx]

**Response to Reviewers**

Revised Manuscript with Track Changes

Manuscript

Thank you very much for the work of the reviewers and editors, which will increase the interest of the manuscript. You will find below a point-by-point answer to each of the reviewers’ and editor’s comments and the indications of changes made to the manuscript.

Reviewers' comments:

Reviewer #1: Well-written article. I have some minor typographical/grammatical suggestions:

Non-inclusion criteria = exclusion criteria?

Trough = through?

Given we included = given the included?

they is a metric = they are?

Please check for dots and comma’s in tables

**Sorry for these mistakes, we have corrected them.**

Reviewer #2: The authors described in this review and metaanalysis the prognostic impact of microcirculation damage measured invasively during STEMI. They conclude that both the IMR and the HMR, (2 quantifiable markers of damage to the microcirculation) considered qualitatively have a prognostic interest.

I have a few minor points to discuss or clarify in the introduction, method, discussion and limitations/

- In the abstract, please correct 2019 as a study published in 2020 was included in the metaanalyses.

**We have corrected date in abstract.**

- Authors should justify the threshold value choosen for IMR and HMR to define severe CMVD.

**We completed the manuscript to answer of this question: “according the literature, it appeared that the IMR and HMR thresholds for the diagnosis of severe CMVD related to worse prognosis varied among teams. To standardize and stay around the most represented thresholds, severe CMVD was defined by a post-PCI IMR > 40mmHg/s (+/- 10%) or a HMR > 3 mmHg/cm/sec (+/- 10%)”.**

- Could the authors provide some data regarding the location of STEMI patients, the culprit artery? Were IMR and HMR measured on the infarted area?

**For all studies, IMR and HMR were performed on infarct-related artery. According the 1094 patients of meta-analysis, 551 (50.5%) infarct-related arteries were left anterior descending arteries (LAD), 132 (12%) were left circumflex (LCx), 406 (37%) were right coronary arteries (RCA), and 5 (0.5%) were left main coronary artery (LM). Jin et al (10) presented a distribution was as follows: LAD 66%, LCx 6%, and RCA 28%. For Carrick et al (13), distribution was: LAD 37%, LCx 18%, RCA 43%, and LM 2%. DeWaard et al (14) had this distribution: LAD 63%, LCx 10%, and RCA 27%. The distribution of Fearon et al (15) was: LAD 55%, LCx 9%, and RCA 36%. Fukunaga et al (16) presented the distribution as follows: LAD 52%, ,LCx 9%, and RCA 39%. Finally, for Maznyczka et al (17), distribution was: LAD 37%, LCx 46%, and RCA 17%.**

- Authors should discuss the use of other potential surrogates of microcirculation disease (TIMI flow, MVO for example) and acknowledge the lack of such angiographic or imaging data in the considered studies.

**Several surrogates of microcirculation disease were used in the considered studies.
We have added these elements in results session and discussion session:**

**“Several others surrogate for microcirculatory disease were used in these studies as Thrombolysis In Myocardial Infarction (TIMI) flow grade [10, 13-17], corrected TIMI frame count (TFC) [13-17], myocardial perfusion grade (MPG) [13,17], and microvascular obstruction (MO) [13,14,16,17]”.**

**“It should be noted that in the studies presented in this meta-analysis other potential surrogates for microcirculatory disease (TIMI, TFC, MPG myocardial perfusion grade, MVO) were used but appeared to provide lower prognostic performance in comparison to IMR or HMR”.**

- In the discussion the following sentence should be referenced or removed "The assessment of HMR is probably more challenging than that of IMR, with higher failure rates related to unreliable doppler flow velocity tracings"

**We have referenced the sentence: Barbato E, Aarnoudse W, Aengevaeren WR, et al. Validation of coronary flow reserve measurements by thermodilution in clinical practice. Eur Heart J. 2004;25:219-23**

Reviewer #3: In their meta-analysis, Canu et al. aimed to conduct a systematic review of the existing literature related to the clinical implications of severe CMVD in STEMI patients. This study is well design, original and very interesting.

The main limitation is the limited number of eligible studies, but the authors emphasized this in their discussion. However, the study provides interesting insights that deserve to be published.

**Thank you for these comments.**
